# Visible-wavelength vectorial holography based on an MIM metasurface

**DOI:** 10.1186/s11671-026-04696-2

**Published:** 2026-06-09

**Authors:** Yin Zhou, Rui Miao, Zihan Geng, Mu Ku Chen

**Affiliations:** 1 Department of Electrical Engineering, City University of Hong Kong, Kowloon, 999077 Hong Kong China; 2 Department of Electrical Engineering, City University of Hong Kong (Dongguan), Dongguan, 523808 Guangdong China; 3 Institute of Data and Information, Tsinghua Shenzhen International Graduate School, Tsinghua University, Shenzhen, 518071 Guangdong China; 4 State Key Laboratory of Terahertz and Millimeter Waves, City University of Hong Kong, Kowloon, 999077 Hong Kong China; 5 City University of Hong Kong Shenzhen Research Institute, Shenzhen, 518057 China

**Keywords:** Vectorial holography, Metasurface, Polarization distributions

## Abstract

Holography plays a vital role in optics, while conventional methods suffer from complex devices and bulky systems. Metasurfaces, versatile platforms for manipulating light at subwavelength scales, have enabled the realization of compact holographic devices. However, most existing metasurface holograms are limited to scalar fields, modulating only intensity while neglecting polarization information. While vectorial holography has attracted significant attention, particularly utilizing metal–insulator-metal (MIM) structures, achieving high-performance vectorial manipulation at visible wavelengths remains challenging due to high ohmic losses. Here, we demonstrate visible-wavelength vectorial holography using an MIM metasurface. Our approach combines an improved vectorial Gerchberg-Saxton (GS) algorithm with a wave-decomposition technique to design subwavelength meta-atoms. We perform full-wave simulations to demonstrate that it can generate target vectorial holographic images under illumination by circularly polarized light at 633 nm, providing an implementation case for vectorial light-field manipulation in the visible wavelength and demonstrating the potential for realizing advanced vectorial holographic displays in the visible wavelength using standard MIM structures.

## Introduction

Holography is a crucial optical technique for recording and reconstructing target light fields. In recent years, it has garnered widespread attention for its applications in virtual reality, structured light, data encryption, and optical storage [1–6]. While traditional holography often relies on complex optical setups, the emergence of metasurfaces combined with the Gerchberg–Saxton (GS) algorithm offers a compact solution.

Metasurfaces, dense arrangements of subwavelength scatterers in a plane, have been demonstrated to enable control of light fields in amplitude, phase, and polarization [7]. Metasurfaces have enabled numerous remarkable effects, including structural color [8,9], electromagnetic invisibility [10,11], depth sensing [12,13] and so on [14–16]. In particular, by arranging meta-atoms with reflection or transmission phases that match the phase distribution iteratively generated by the GS algorithm, a metasurface can be constructed to generate pre-designed holographic images under designed incident light [17–20]. Compared to conventional holographic methods, metasurface-based holography offers greater design flexibility, larger diffracting angles, easier integration, and greater miniaturization. However, most existing metasurface-based approaches are limited to the scalar regime. Although some metasurface designs employ polarization multiplexing to switch images between distinct polarization states, they typically exhibit uniform polarization distributions across the image, failing to manipulate the polarization state pixel-by-pixel [21–23]. Achieving efficient vectorial holography represents a critical frontier for advancing higher-dimensional light field control. It can be extended to broader application scenarios, such as polarized illumination, optical encryption, anticounterfeiting, and data storage [24,25]. However, conventionally, vectorial holography requires cascading dynamic modulators (e.g., SLMs) combined with interferometric setups to control orthogonal polarization channels independently. Such systems are inherently bulky, sensitive to vibrations, and difficult to align. In contrast, metasurfaces offer a route to integrate these functionalities into a single, ultrathin interface.

Driven by this potential, efforts have been made to generate vectorial holographic images using metasurfaces [26–30]. Nevertheless, these approaches each have their own limitations, such as restricting specific incident light, requiring the use of multiple layers of metasurfaces, exhibiting low efficiency, or only achieving discrete polarization distributions. Recently, a highly efficient generic strategy for designing vectorial holograms has been proposed to generate pre-designed vector holographic images under arbitrary incident polarization with cross-shaped meta-atoms based on a metal–insulator-metal (MIM) structure [31,32].

In this work, we demonstrate an approach that combines the improved vectorial GS algorithm and the wave-decomposition technique to iteratively design the metasurface phase profile. We utilize cross-shaped meta-atoms that exhibit reflection phases of both the Pancharatnam-Berry (PB) phase and resonant phase under visible light. Based on this design, we performed full-wave simulations of the metasurfaces, demonstrating their ability to generate target complex vector holographic images under 633 nm incident light. It achieves direct compatibility with existing mainstream visible-light optical systems, opening new avenues for its application in polarized illumination, optical encryption, and other fields.

## Results

### Generic strategy for designing vectorial holograms

**Fig. 1:**
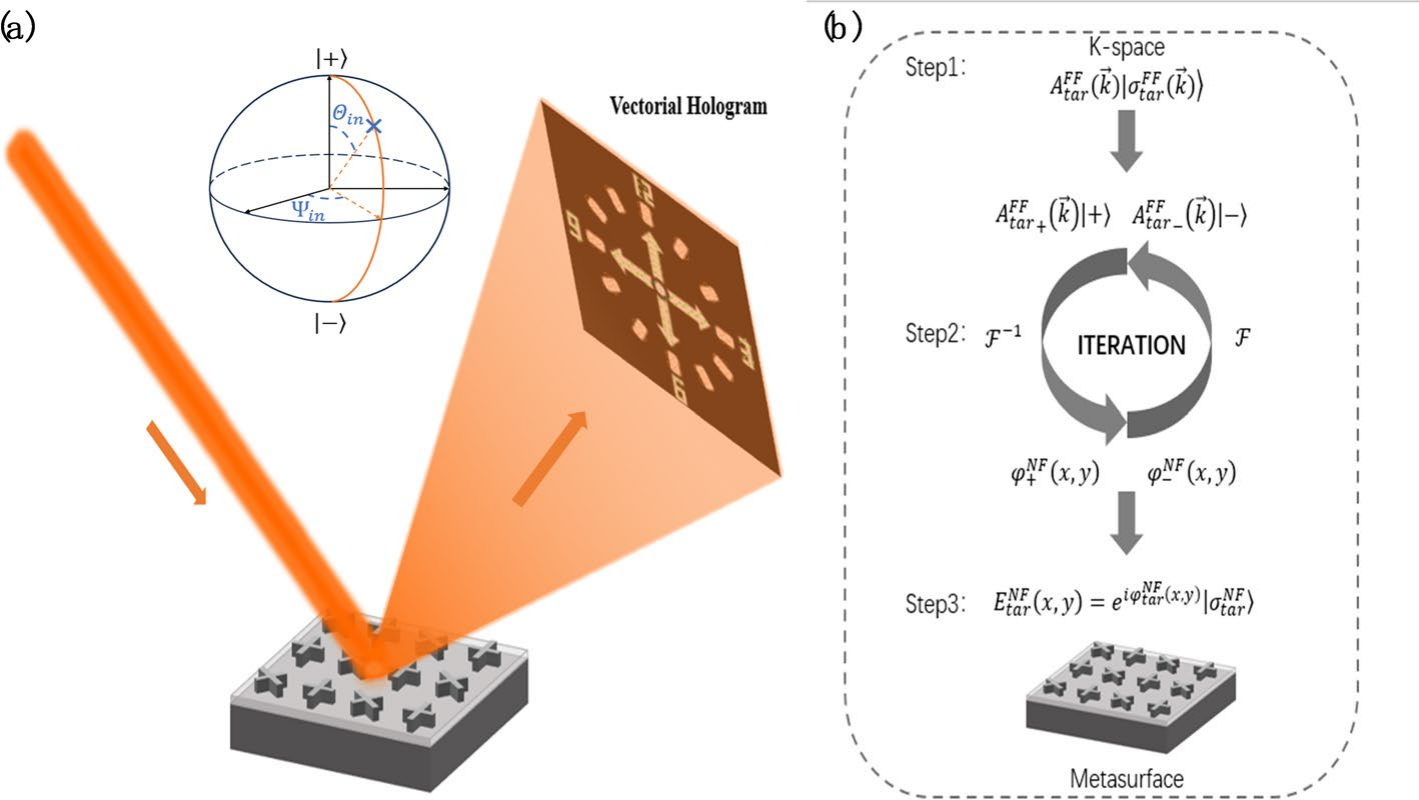
Schematic diagram of metasurface vectorial holograms and their general design process. **a** When incident light illuminates a metasurface with a customized reflective phase and polarization-conversion capabilities, a vectorial hologram can be generated in the far field. **b** Flow chart of the general process

We employ a generic strategy to design a metasurface for a vectorial hologram. We assume a metasurface, when it is illuminated by a plane wave incident light with specific polarization $$|{\sigma}_{in}\rangle =\;\left(\begin{array}{c}{e}^{-i{\Psi}_{in}/2}cos({\Theta}_{in}/2)\\ {e}^{+i{\Psi}_{in}/2}sin({\Theta}_{in}/2)\end{array}\right)$$, can generate a target vectorial hologram in the far-field (FF). In particular, the polar angle ${\Theta}_{in}$ and the azimuthal angle ${\Psi}_{in}$ denoting its position on Poincaré’s sphere as shown in Fig. 1a. To describe the vector beam in the FF, it is customized to set targets in the $\overrightarrow{k}$-space. Here, we use ${A}_{tar}^{FF}(\overrightarrow{k})$ and $|{\sigma}_{tar}^{FF}(\overrightarrow{k})\rangle$ to express the beam's complex amplitude and polarization state. In this situation, ${\left|{A}_{tar}^{FF}(\overrightarrow{k})\right|}^{2}$ and $|{\sigma}_{tar}^{FF}(\overrightarrow{k})\rangle$ describe the intensity and polarization information of the field distribution measured on the focal plane of a lens, which is a common method for obtaining vector beam images in FF characterization experiments [31]. The target for the algorithm is to iterate the NF distributions of the metasurface through the desired ${\left|{A}_{tar}^{FF}(\overrightarrow{k})\right|}^{2}$ and $|{\sigma}_{tar}^{FF}(\overrightarrow{k})\rangle$. The whole strategy can be divided into three steps as it shown in Fig. 1b. Firstly, decomposing the FF vector beam into a left-handed circularly polarized (LCP) beam and a right-handed circularly polarized (RCP) beam. Secondly, using the GS algorithm to generate the two NF distributions required to produce these two FF beams. At last, constructing the final vectorial NF distribution via the superposition of the two NF distributions. That’s the final target NF distribution we should achieve via metasurfaces.

In detail, firstly, the target polarization distribution in FF can be written as $$|{\sigma}_{tar}^{FF}(\overrightarrow{k})\rangle =\left(\begin{array}{c}{e}^{-i{\Psi}_{tar}^{FF}(\overrightarrow{k})/2}\mathrm{cos}({\Theta}_{tar}^{FF}(\overrightarrow{k})/2)\\ {e}^{+i{\Psi}_{tar}^{FF}(\overrightarrow{k})/2}\mathrm{sin}({\Theta}_{tar}^{FF}(\overrightarrow{k})/2)\end{array}\right)$$. The polar angle ${\Theta}_{tar}^{FF}$ and the azimuthal angle ${\Psi}_{tar}^{FF}$ denoting its specific position on Poincaré’s sphere, representing the target polarization state in FF. Under the paraxial approximation, the design and analysis of vector beams can be simplified from complex vector coupling problems to the design and superposition of two scalar fields. Therefore, it can be decoupled into two CP bases, ${{A}_{tar}^{FF}}_{+}(\overrightarrow{k})|+\rangle \;and\; {{A}_{tar}^{FF}}_{-}(\overrightarrow{k})|-\rangle$, where $|+\rangle$ and $|-\rangle$ represent LCP and RCP, respectively. These two beams interfere with an appropriate phase difference $arg({{A}_{tar}^{FF}}_{-}(\overrightarrow{k}))-arg({{A}_{tar}^{FF}}_{+}(\overrightarrow{k}))=\Delta {\Phi}_{tar}^{FF}(\overrightarrow{k})={\Psi}_{tar}^{FF}(\overrightarrow{k})$, exhibiting the target image with an arbitrary polarization distribution. We have Eq. (1) to express the two complex amplitudes as

1 $$\begin{array}{c}\left\{\begin{array}{c}{{A}_{tar}^{FF}}_{+}\left(\overrightarrow{k}\right)={A}_{tar}^{FF}\left(\overrightarrow{k}\right){e}^{- \frac{i{\Psi}_{tar}^{FF}\left(\overrightarrow{k}\right)}{2}}\mathrm{cos}\left(\frac{{\Theta}_{tar}^{FF}\left(\overrightarrow{k}\right)}{2}\right)\\ {{A}_{tar}^{FF}}_{-}\left(\overrightarrow{k}\right)={A}_{tar}^{FF}\left(\overrightarrow{k}\right){e}^{+ \frac{i{\Psi}_{tar}^{FF}\left(\overrightarrow{k}\right)}{2}}\mathrm{sin}\left(\frac{{\Theta}_{tar}^{FF}\left(\overrightarrow{k}\right)}{2}\right)\end{array}\right.\end{array}$$

Next, we use the GS algorithm to iteratively retrieve the NF distribution at the meta-plane (z = 0). The NF phase distribution ${\varphi}_{+}^{NF}\left(x,y\right)$ of LCP is iterated from the designed FF intensity distribution ${I}_{+}(\overrightarrow{k}) = {\left|{{A}_{tar}^{FF}}_{+}(\overrightarrow{k})\right|}^{2}$ of LCP, while the NF phase distribution ${\varphi}_{-}^{NF}\left(x,y\right)$ of RCP is iterated from the designed FF intensity distribution ${I}_{-}(\overrightarrow{k}) = {\left|{{A}_{tar}^{FF}}_{-}(\overrightarrow{k})\right|}^{2}$ of RCP. It is worth mentioning that, during the iteration process, the complex amplitude phase difference $\Delta {\Phi}_{tar}^{FF}(\overrightarrow{k})$ between the two sets of polarized light is constrained to remain constant, ensuring that their final FF polarization states will not change during the iteration.

**Fig. 2:**
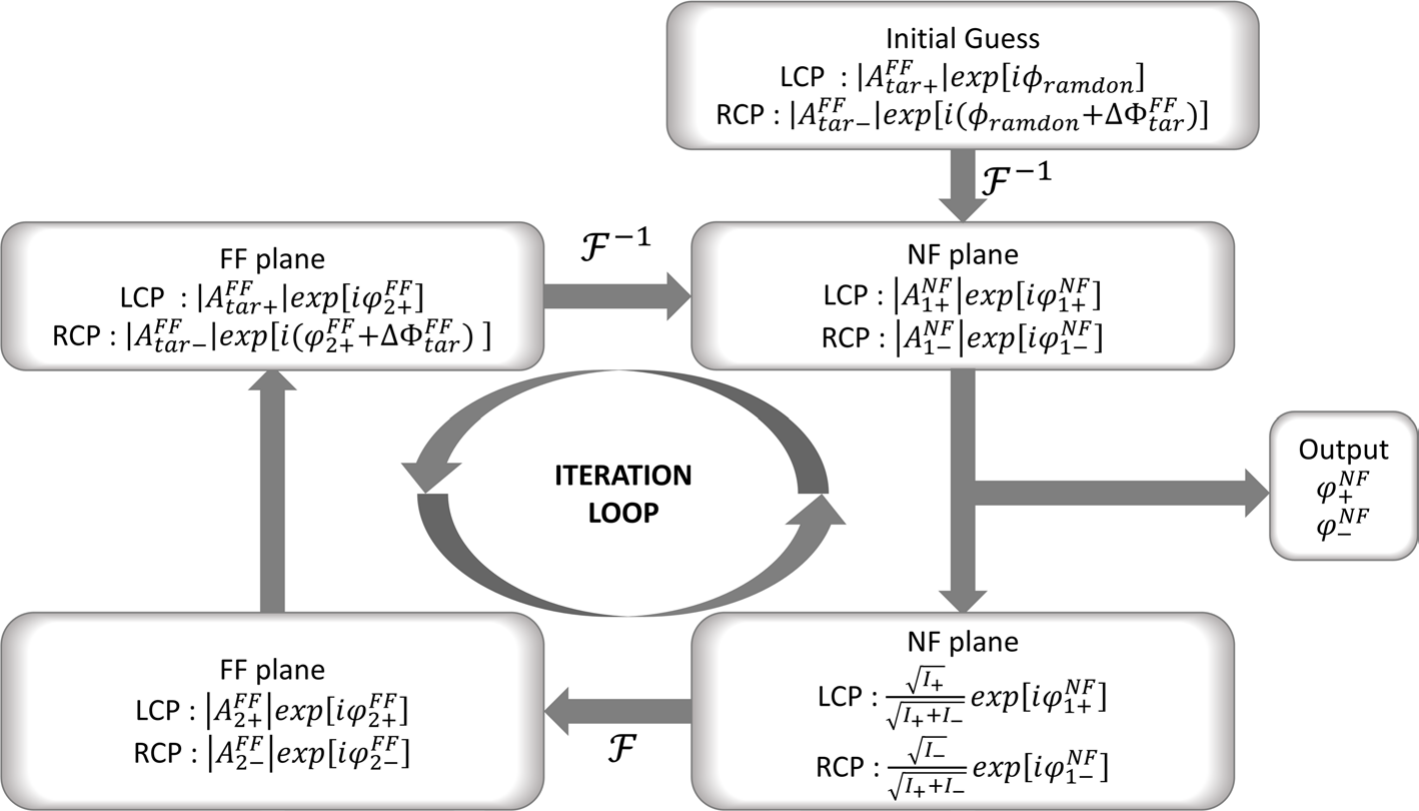
The flow chart of the modified vectorial GS algorithm

At last, we have to build the final NF distribution via the iterated two CP-based NF distribution. However, the classic GS algorithm can only derive the NF phase distribution, failing to get amplitude information. Therefore, in the method, to maintain conservation of energy, setting the amplitudes of LCP and RCP as $\sqrt{\frac{{I}_{+}}{{I}_{+}+{I}_{-}}}$ and $\sqrt{\frac{{I}_{-}}{{I}_{+}+{I}_{-}}},$ respectively. Where ${I}_{+}$ and ${I}_{-}$ represents intensities of LCP and RCP components in FF, which can be calculated via Eq. (2). Figure 2 describes the whole algorithm for this GS algorithm. Building on the established wave-decomposition Gerchberg–Saxton (GS) framework, we first demonstrate full-vectorial holographic control at 633 nm (visible wavelength). While previous implementations of this algorithm have been confined to the infrared band, we extend its applicability to the visible regime, despite the increased ohmic losses inherent to the MIM structure at this wavelength. Through systematic parameter optimization, we identified suitable structural configurations that enable simultaneous manipulation of left- and right-circularly polarized components with prescribed amplitude and phase relationships, thereby achieving high-fidelity full-vectorial holography in the visible band for the first time.

2 $$\begin{array}{c}\left\{\begin{array}{c}{I}_{+}={\sum}_{\overrightarrow{k}}{\left|{{A}_{tar}^{FF}}_{+}\left(\overrightarrow{k}\right)\right|}^{2} \\ {I}_{-}= {\sum}_{\overrightarrow{k}}{\left|{{A}_{tar}^{FF}}_{-}\left(\overrightarrow{k}\right)\right|}^{2}\end{array}\right.\end{array}$$

Therefore, the final target NF distribution at meta-plane can be written as

3 $$\begin{array}{c}{E}_{NF}^{tar}\left(x,y\right)= \sqrt{\frac{{I}_{+}}{{I}_{+}+{I}_{-}}}{e}^{i{\varphi}_{+}^{NF}\left(x,y\right)}|+\rangle +\sqrt{\frac{{I}_{-}}{{I}_{+}+{I}_{-}}}{e}^{i{\varphi}_{-}^{NF}\left(x,y\right)}|-\rangle \\ ={e}^{i{\varphi}_{tar}^{NF}\left(x,y\right)}\left(\genfrac{}{}{0pt}{}{{e}^{- \frac{i{\Psi}_{tar}^{NF}\left(x,y\right)}{2}\mathrm{cos}\left(\frac{{\Theta}_{tar}^{NF}\left(x,y\right)}{2}\right)}}{{e}^{+ \frac{i{\Psi}_{tar}^{NF}\left(x,y\right)}{2}\mathrm{sin}\left(\frac{{\Theta}_{tar}^{NF}\left(x,y\right)}{2}\right)}}\right)={e}^{i{\varphi}_{tar}^{NF}\left(x,y\right)}|{\sigma}_{tar}^{NF}\rangle \end{array}$$

where

4 $$\begin{array}{c}\left\{\begin{array}{c}{\varphi}_{tar}^{NF}\left(x,y\right)= \frac{{\varphi}_{+}^{NF}\left(x,y\right)+{\varphi}_{-}^{NF}\left(x,y\right)}{2}\\ {\Psi}_{tar}^{NF}\left(x,y\right)={\varphi}_{-}^{NF}\left(x,y\right)-{\varphi}_{+}^{NF}\left(x,y\right)\\ {\Theta}_{tar}^{NF}\left(x,y\right)=2*\mathrm{arctan}\left(\frac{\sqrt{{I}_{-}}}{\sqrt{{I}_{+}}}\right)\end{array}\right.\end{array}$$

It characterizes the NF distribution on the meta-plane at position (x,y) with phase ${\varphi}_{tar}^{NF}\left(x,y\right)$ and a polarization state $|{\sigma}_{tar}^{NF}(x,y)\rangle =\left(\genfrac{}{}{0pt}{}{{e}^{- \frac{i{\Psi}_{tar}^{NF}\left(x,y\right)}{2}\mathrm{cos}\left(\frac{{\Theta}_{tar}^{NF}\left(x,y\right)}{2}\right)}}{{e}^{+ \frac{i{\Psi}_{tar}^{NF}\left(x,y\right)}{2}\mathrm{sin}\left(\frac{{\Theta}_{tar}^{NF}\left(x,y\right)}{2}\right)}}\right).$ $\left\{{\varphi}_{tar}^{NF}{,\Psi }_{tar}^{NF}{,\Theta }_{tar}^{NF}\right\}$ denoting its position on Poincaré’s sphere, representing the required polarization state and phase in the NF.

### Design of meta-atoms

**Fig. 3:**
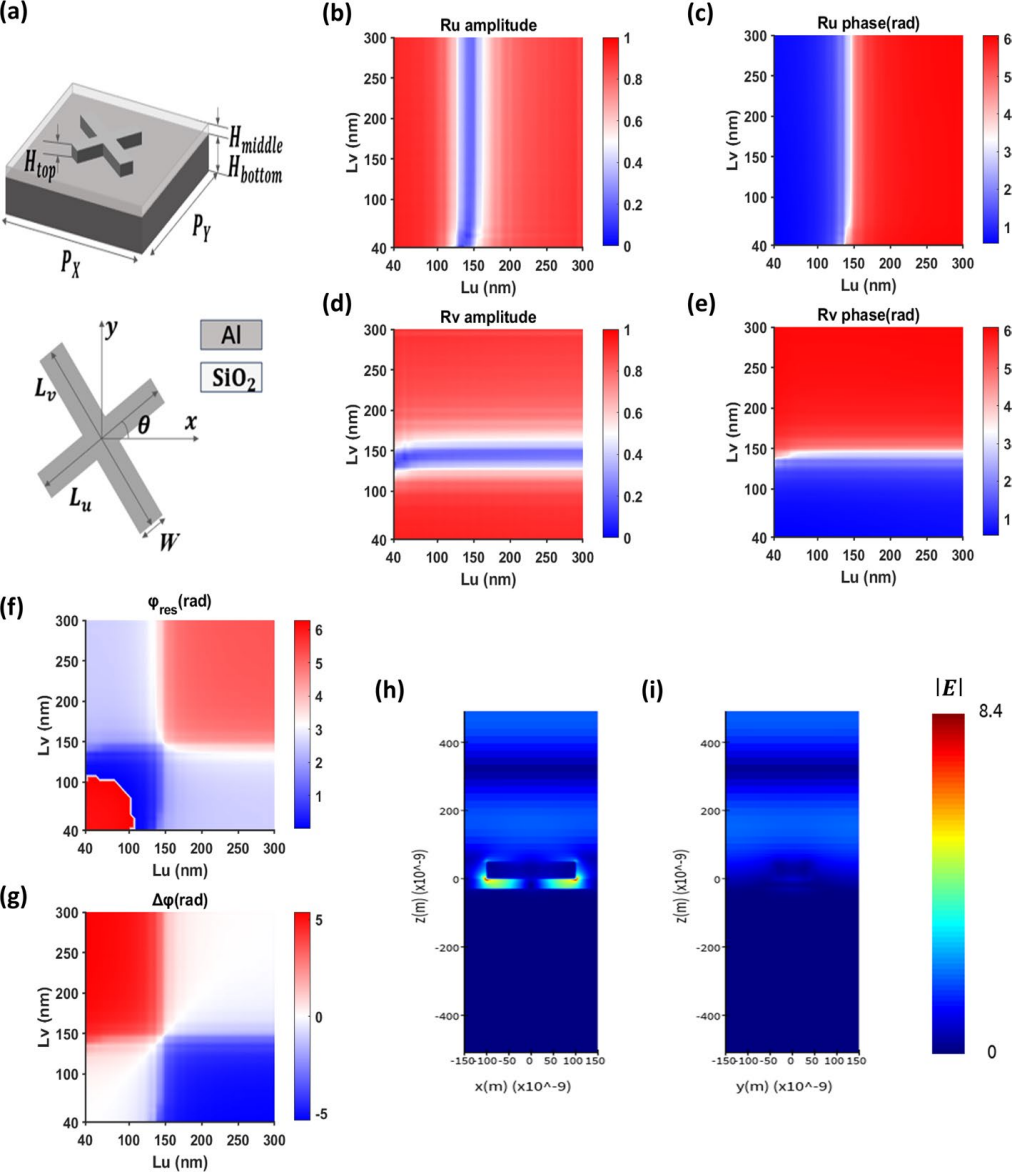
Structure and characterizations of meta-atoms. **a** Structure of the meta-atom based on MIM structure. **b** Ru amplitude $\left|{r}_{uu}\right|$, **c** Ru phase ${\varphi}_{u}$, **d** Rv amplitude $\left|{r}_{vv}\right|$, **e** Rv phase ${\varphi}_{v}$, **f** Average resonance phase ${\varphi}_{res}$ and **g** cross-polarization phase difference $\Delta \varphi$ of meta-atoms arrays vary with different ${L}_{u}$ and ${L}_{v}$, calculated by FDTD simulations under 633 nm. Other geometry parameters are fixed as *H*_*top*_ = 50 nm, *H*_*middle*_ = 30 nm, *H*_*bottom*_ = 170 nm, *W* = 40 nm, *θ* = 0*°*, and *P*_*X*_ = *P*_*Y*_ = 300 nm. The electric field distributions along the **h** u-axis and **i** v-axis, respectively

The target is to generate this NF distribution via a metasurface and a designed incident light with a specific polarization state. The meta-atoms are supposed to have a required phase ${\varphi}_{tar}^{NF}\left(x,y\right)$ and the ability to transfer the polarization state $|{\sigma}_{in}\rangle$ to $|{\sigma}_{tar}^{NF}\rangle$. According to previous works, a meta-atom with metal–insulator-metal (MIM) structure will be a suitable choice for this [17,21,31–33]. While ohmic losses are inevitable in all metals in the visible spectrum, aluminum (Al) forms a stable, dense oxide layer that prevents an increase in losses. Although this oxide layer does have some impact on its performance, it also prevents further degradation of the Al. Through rigorous structural optimization of the Al-based metal–insulator–metal architecture, a phase library capable of achieving vectorial holography is constructed. The detailed structure can be seen in Fig. 3a. It is composed of a cross-shaped aluminum layer on top, a silicon dioxide intermediate layer in the middle, and a bottom metal film (aluminum). When the meta-atom is illuminated by incident light polarized along the u-axis (or v-axis) of the E-field, counter-current currents are induced in the two metal layers, forming a magnetic resonance whose resonance frequency is determined by L_u_ (or L_v_) (from 40 to 300 nm). In the MIM structure, the mode that triggers the response is a gap plasmon induced by the thin SiO_2_ layer, and the electric field is locally confined within the gap. Due to the metal film on the bottom, it exhibits high reflectivity in most cases. In the u − v coordinate system, its reflectance Jones matrix can be expressed as J = $$\left(\begin{array}{cc}\left|{r}_{uu}\right|{e}^{i{\varphi}_{u}}& 0\\ 0& \left|{r}_{vv}\right|{e}^{i{\varphi}_{v}}\end{array}\right)$$, where $\left|{r}_{uu}\right|{e}^{i{\varphi}_{u}}$ and $\left|{r}_{vv}\right|{e}^{i{\varphi}_{v}}$ are the complex reflectance coefficients along the u-axis and v-axis of meta-atoms, respectively. Here, for simplicity, only phases are considered in further analysis.

Here, two new parameters are defined, ${\varphi}_{res}= {(\varphi }_{u}+{\varphi}_{v})/2+\frac{\pi }{4}$ and $\Delta \varphi ={\varphi}_{v}-{\varphi}_{u}$, representing the average resonance phase and the cross-polarization phase difference, respectively. Figure 3b–g shows its characterizations varying with its geometry parameters, L_u_ and L_v_. The results were simulated at the wavelength of 633 nm with fixed other geometry parameters: ${H}_{\mathrm{top}}= 50\;\mathrm{nm}, {H}_{\mathrm{middle}} = 30\;\mathrm{nm}, {H}_{\mathrm{bottom}}= 170\;\mathrm{nm}, W=40\;\mathrm{nm},{P}_{X}={P}_{Y}=300\;\mathrm{nm}$. Allium (Al) is chosen to be the metal layer, and silicon dioxide (SiO_2_) is chosen to be the insulator layer.

The reflection Jones matrix can be written in the circularly polarized basis as ${J}_{CP}=QR(\theta )JR(-\theta ){Q}^{-1}$,where $Q$ represents LP to CP transformation matrix and R($\theta$) = $$\left(\begin{array}{cc}\mathrm{cos}\theta & -\mathrm{sin}\theta \\ \mathrm{sin}\theta & \mathrm{cos}\theta \end{array}\right)$$ represents the rotation matrix. With an incident polarization light $$|{\sigma}_{in}\rangle =\;\left(\begin{array}{c}{e}^{-i{\Psi}_{in}/2}\mathrm{cos}({\Theta}_{in}/2)\\ {e}^{+i{\Psi}_{in}/2}\mathrm{sin}({\Theta}_{in}/2)\end{array}\right)$$, the NF filed distribution after modulation of the meta-atom can be written as

$${E}_{NF}={J}_{CP}|{\sigma}_{in}\rangle $$

5 $$\begin{array}{c}={e}^{i\left({\varphi}_{res}+\frac{\pi }{4}\right)}\left(\genfrac{}{}{0pt}{}{{e}^{-i\frac{{\Psi}_{in}}{2}}\mathrm{cos}\frac{{\Theta}_{in}}{2}\mathrm{cos}\frac{\Delta \varphi }{2}-i{e}^{i-2\theta }{\mathrm{sin}\frac{\Delta \varphi }{2}*e}^{+i\frac{{\Psi}_{in}}{2}}\mathrm{sin}\frac{{\Theta}_{in}}{2}}{{e}^{+i\frac{{\Psi}_{in}}{2}}\mathrm{sin}\frac{{\Theta}_{in}}{2}\mathrm{cos}\frac{\Delta \varphi }{2}-i{e}^{i2\theta }\mathrm{sin}\frac{\Delta \varphi }{2}{*e}^{-i\frac{{\Psi}_{in}}{2}}\mathrm{cos}\frac{{\Theta}_{in}}{2}}\right)\end{array}$$

It’s worth to mention that it still satisfies energy conservation. Besides, cases under incident light with different ${\Psi}_{in}$ can be treated identically by setting the starting rotating angle of the meta-atom to ${\Psi}_{in}$/2 to compensate (The meta-atoms possess C2 symmetry.). As a result, it’s reasonable to assume ${\Psi}_{in}$=0 to simplify. Through Eq. (3) and (5), the relationship between $\left\{{\varphi}_{tar}^{NF}{,\Psi }_{tar}^{NF}{,\Theta }_{tar}^{NF}\right\}$ and $\left\{{\varphi}_{res},\Delta \varphi ,\theta ,\Theta \right\}$ can be calculated as

6 $$\begin{array}{c}\left\{\begin{array}{c}{\varphi}_{tar}^{NF}= {\varphi}_{res}+\frac{\pi }{4}+\frac{\mathrm{arg}\left(A\right)+\mathrm{arg}\left(B\right)}{2}\\ {\Psi}_{tar}^{NF}=\mathrm{arg}\left(B\right)-\mathrm{arg}\left(A\right)\\ {\Theta}_{tar}^{NF}=2*arctan\frac{\left|B\right|}{\left|A\right|}\end{array}\right.\end{array}$$

where A represents $\left(\mathrm{cos}\frac{{\Theta}_{in}}{2}\mathrm{cos}\frac{\Delta \varphi }{2}-i{e}^{i-2\theta }\mathrm{sin}\frac{\Delta \varphi }{2}\mathrm{sin}\frac{{\Theta}_{in}}{2}\right)$ and B represents $\left(\mathrm{sin}\frac{{\Theta}_{in}}{2}\mathrm{cos}\frac{\Delta \varphi }{2}-i{e}^{i2\theta }\mathrm{sin}\frac{\Delta \varphi }{2}\mathrm{cos}\frac{{\Theta}_{in}}{2}\right)$. In fact, these three relationships will be very complex, but they will become the simplest when we choose LCP (Ψ = 0, Θ = 0) as the incident light, as shown in Eq. (7).

7 $$\begin{array}{c}\left\{\begin{array}{c}{\varphi}_{tar}^{NF}\left(x,y\right)={\varphi}_{res}+\theta \\ {\Theta}_{tar}^{NF}\left(x,y\right)=\Delta \varphi \\ {\Psi}_{tar}^{NF}\left(x,y\right)=2\theta -\frac{\pi }{2}\end{array}\right.\end{array}$$

According to Eqs. (4), (7), and the final relationship between the CP basis phase, ${\varphi}_{+}^{NF}\left(x,y\right)$ and ${\varphi}_{-}^{NF}\left(x,y\right)$ retrieved from the GS algorithm, the average resonance phase ${\varphi}_{res}$ and cross-polarization phase difference $\Delta \varphi$ from simulations, and rotating angles of meta-atoms $\theta$ will be determined as

8 $$\begin{array}{c}\left\{\begin{array}{c}{\varphi}_{res}(x,y)= {\varphi}_{+}^{NF}\left(x,y\right)-\frac{\pi }{4}\\ \Delta \varphi (x,y)=2*\mathrm{arctan}\left(\frac{\sqrt{{I}_{-}}}{\sqrt{{I}_{+}}}\right)\\ \theta (x,y)=\frac{{\varphi}_{-}^{NF}\left(x,y\right)-{\varphi}_{+}^{NF}\left(x,y\right)}{2}+\frac{\pi }{4}\end{array}\right.\end{array}$$

Based on this, the arrangement of metasurfaces can be determined, thereby achieving full-vectorial holograms.

An assessment of potential nanofabrication tolerances is discussed. As shown in the updated Fig. 3f and g, the phase response exhibits a relatively flat gradient over most of the designed size ranges, indicating a high tolerance to lateral fabrication errors such as L_u_ and L_v_. Meanwhile, the GS algorithm is highly robust and exhibits some tolerance to phase discrepancy. However, we acknowledge that the resonance in the MIM structure is sensitive to the thickness of the dielectric layer. Fortunately, modern deposition techniques such as Atomic Layer Deposition (ALD) and RF sputtering can control thickness with sub-nanometer precision, which can partly solve this problem [34,35].

The electric field distributions along the u-axis and v-axis are shown in Fig. 3h,i, which show that the mode is a gap plasmon induced by the thin SiO_2_ layer. The results show that the electric field is strongly localized in the metal–insulator-metal gap region (from z = − 30 nm to 0 nm), consistent with the characteristics of a finite-length MIM gap plasmon cavity. Meanwhile, under x-polarized incident light, the electric field along the y-axis shows little response, indicating extremely low crosstalk between the two orthogonal polarization channels.

For the potential fabrication process, the MIM structure can be fabricated using standard thin-film deposition and EBL techniques. Firstly, a 170 nm-thick aluminum (Al) layer (via magnetron DC sputtering) and a 30 nm-thick SiO_2_ layer (via RF sputtering) are deposited onto a silicon substrate in a sputtering system without breaking vacuum, thereby preventing the Al layer from forming a dense oxide film. Then, a 100 nm-thick layer of photoresist is coated on the SiO_2_ layer. Using electron-beam lithography (EBL), cross-shaped structures are formed after development, and a 50 nm-thick Al layer is then deposited on them. Finally, the Al cross-shaped structures are successfully fabricated via lift-off.

### Results of simulation

**Fig. 4:**
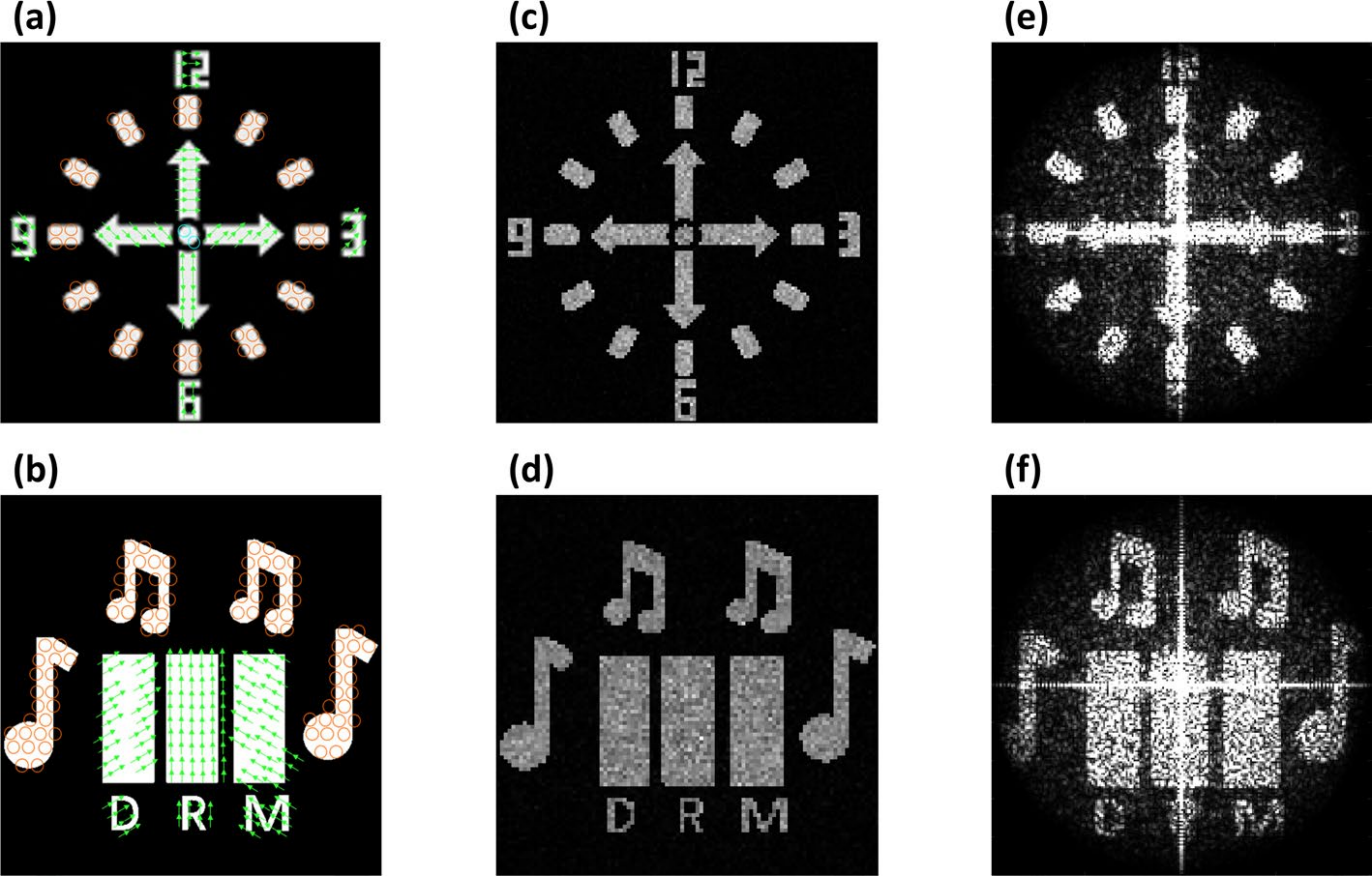
Target holographic Intensity and polarization distribution of **a** the first image and **b** the second image. Algorithm-derived FF intensity results of **c** the first image and **d** the second image. FDTD simulated FF intensity results of **e** the first image and **f** the second image. In the first image, the numbers 12, 3, 6, and 9, and the corresponding arrows pointing to them, have linear polarization states of 0°, 45°, 90°, and 135°, respectively. In the second image, the letters D, R, M, and the piano keys above them have polarization states of 30°, 90°, and 150°, respectively. The red circle represents LCP, and the blue circle represents RCP

Following the design process outlined in the previous two sections, the algorithm is employed to retrieve the desired phase distribution from the target vector image. We simulated two metasurfaces for two vectorial holograms in FDTD based on the strategy. The FDTD-simulated metasurface comprised 100 × 100 pixels. The two target vectorial images are shown in Fig. 4a,b. The red circle represents LCP, while the blue circle represents RCP, and the bright green arrow indicates linear polarization, whose angle represents the linear polarization angle. Figure 4c,d describes the intensity distribution generated by the algorithm of the two images, and Fig. 4e,f describes the intensity distribution results calculated at FDTD simulations when the designed two metasurfaces are illuminated by 633 nm left-handed circularly polarized light. The crosses in Fig. 4e,f represent the far-field projection of the zero-order light (unmodulated light). Since the designed metasurface is a rectangular structure, the zero-order-intensity distribution in the far field is the Fourier transform of a rectangular function, that is, a two-dimensional sinc function. Some differences primarily stem from phase discretization and simulation scale.

We define the relative diffraction efficiency as

9 $$\eta =\frac{{I}_{signal}}{{I}_{FF}}\times 100\%,$$

where ${I}_{signal}$ is the total energy in the signal zone of the reconstructed holographic image (excluding the central zero-order light), ${I}_{FF}$ is the total energy over the entire far-field plane. Based on calculations from the far-field intensity distribution derived from FDTD, the relative diffraction efficiencies of the two displayed holograms are 22.22% (clock) and 28.98% (piano), respectively.

Much of the previously advanced vectorial holography research has also suffered from various limitations, such as being restricted to specific incident light or employing spatial division multiplexing, which results in low efficiency and high polarization crosstalk [36,37]. In contrast, the strategy we adopt uses a single meta-atom to achieve full vectorial holography, maintaining low polarization crosstalk while not constraining the incident light. The calculated PER (polarization extinction ratio) is 8.77 dB.

**Fig. 5:**
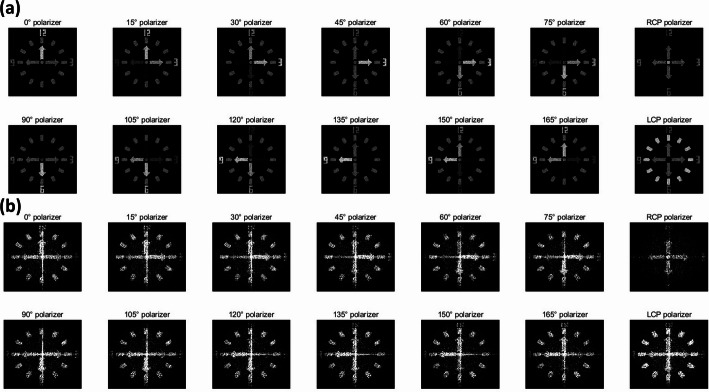
Vector holographic demonstration of clock patterns. **a** Algorithm-derived FF intensity results after polarizers with different angles at ${0}^{^\circ }$–${165}^{^\circ }$(step $15^\circ$), and the RCP polarizer and LCP polarizer of the first image. **b** According to the FDTD-simulated FF intensity results after the polarizers

**Fig. 6:**
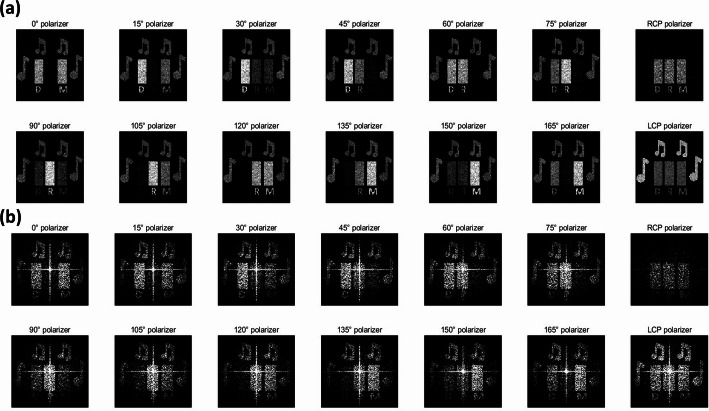
Vector holographic demonstration of musical note patterns. **a** Algorithm-derived FF intensity results after polarizers with different angles at ${0}^{^\circ }$–${165}^{^\circ }$(step $15^\circ$), and the RCP polarizer and LCP polarizer of the first image. **b** According to the FDTD-simulated FF intensity results after the polarizers

Furthermore, to reveal the vectorial characteristics of the generated holographic image, we post-processed the complex amplitude of the electric field, calculated from the algorithm and FDTD simulations, to simulate the transmission of the vectorial hologram through different polarizers. Figure 5a presents the intensity distribution of the first vectorial holographic image generated by the algorithm upon transmission through different polarizers, and Fig. 5b describes the intensity distribution of the first vectorial holographic image calculated at the FDTD simulation upon transmission through different polarizers. Similarly, Fig. 6a presents the intensity distribution of the second vectorial holographic image generated by the algorithm upon transmission through different polarizers, and Fig. 6b describes the intensity distribution of the second vectorial holographic image calculated at the FDTD simulation upon transmission through different polarizers. The results demonstrate great agreement with the designed polarization states.

Notably, Fig. 5 shows that as the polarizer rotates, the vectorial hologram, the clock's hands, and the corresponding Arabic numerals illuminate sequentially. Figure 6 shows that as the polarizer rotates, piano blocks and their corresponding notes exhibit a sequential highlighting effect. This fully demonstrates the additional degrees of freedom introduced by incorporating polarization information into vectorial holography.

To illustrate the influence of the degree of phase discretization and the number of periods on the MIM-based vector hologram, we performed additional FDTD simulations, systematically varying the phase discretization level (4-level vs. 8-level) and the period number (100 × 100 vs. 150 × 150). We evaluated three key parameters: relative diffraction efficiency ($\eta$), polarization extinction ratio (PER), and signal-to-noise ratio (SNR). The results are shown in Table 1. As the phase discretization level and simulation scale increase, all parameters improve.

**Table 1:** Three parameters of different phase discretization levels and array sizes

Arrangements	$\eta$	PER	SNR
4-level, 100 × 100	17.90%	7.17 dB	6.05 dB
8-level, 100 × 100	22.22%	8.77 dB	6.41 dB
8-level, 150 × 150	24.02%	9.05 dB	6.59 dB

**Fig. 7:**
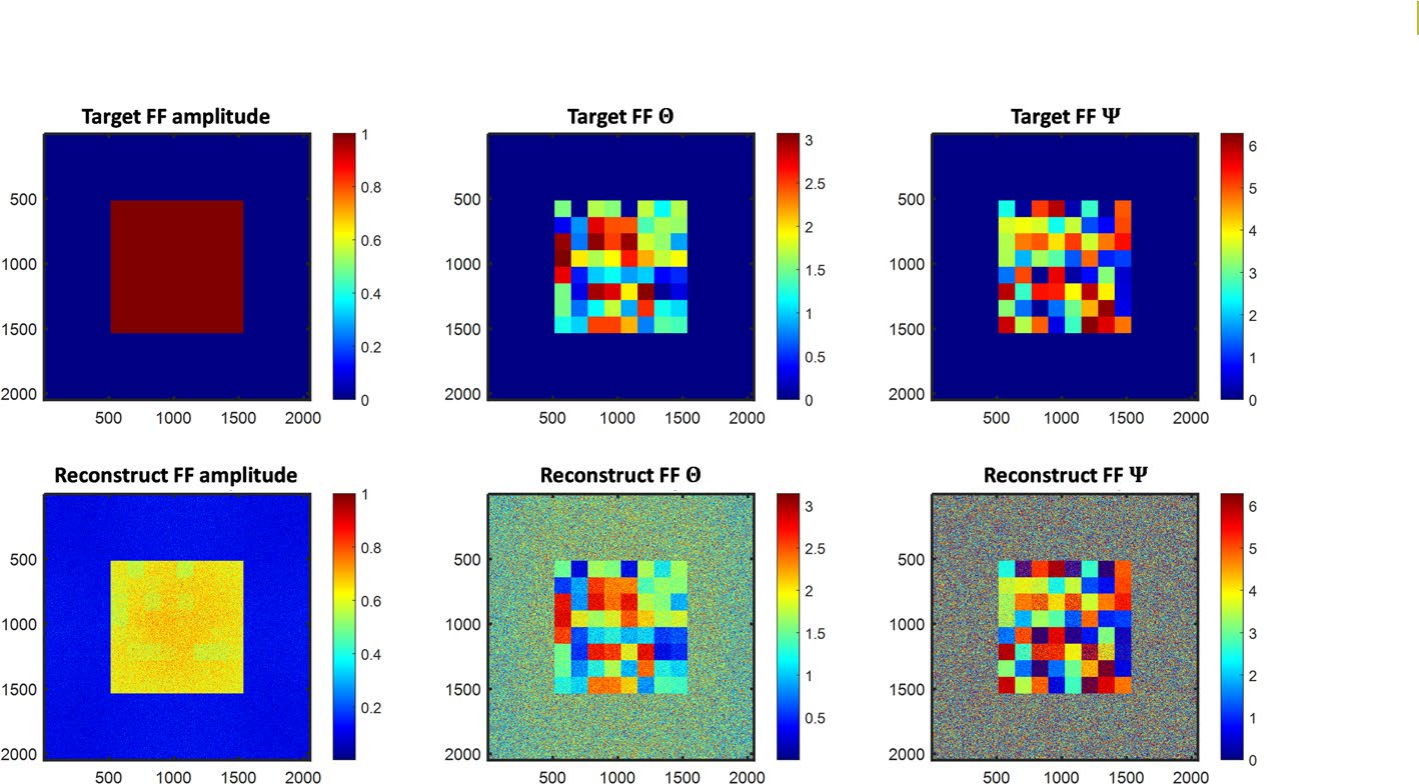
Target and reconstruct the FF distribution with a random polarization distribution

To demonstrate the robustness of MIM-based vector holography, a target holographic image consisting of a large, uniform-amplitude square region in the center (1024*1024 pixels) is defined, as shown in Fig. 7. This large square is subdivided into an array of smaller "boxes", with each adjacent box assigned a distinctly random polarization state. Then, systematically reducing the dimensions of these boxes (from 128 to 8 pixels) and evaluating the Mean Absolute Error (MAE) and Root Mean Square Error (RMSE) of the reconstructed polarization states. The results are shown in Table 2. It is worth noting that in this result, we imposed a strict zero-amplitude constraint on the background (non-signal) region.

**Table 2:** The MAE and RMSE results of different box sizes

Box-size	$MAE$ ($\Theta + \Psi$) (rad)	$RMSE$ ($\Theta + \Psi$) (rad)
128	0.1886 + 0.2733	0.2358 + 0.3812
64	0.1943 + 0.2881	0.2429 + 0.4052
32	0.1965 + 0.2951	0.2456 + 0.4187
16	0.1960 + 0.2929	0.2450 + 0.4145
8	0.1963 + 0.2943	0.2453 + 0.4169

From the table, the MAE and RMSE do not show obvious fluctuations as the box size decreases. Combined with the visual quality of the algorithm's reconstructed results, we believe that most of the error stems from the inevitable speckle of phase-only holography rather than insufficient polarization spatial resolution. To verify this process, readjust the settings. In this algorithm result, we no longer strictly enforce zero amplitude in the non-signal region, which can partially alleviate speckle (This operation introduces significant speckle in the background). The results are shown in Table 3. For all box sizes, both MAE and RMSE are significantly reduced. Even when speckle noise is suppressed, as the box size decreases to 8 pixels, the polarization error shows no significant increase. This clearly demonstrates that the algorithm has extremely high spatial resolution for polarization control. The local polarization state can vary rapidly without suffering significant near-field or far-field crosstalk.

**Table 3:** The MAE and RMSE results of different box sizes (lower speckles in the signal zone)

Box-size	$MAE$ ($\Theta + \Psi$) (rad)	$RMSE$ ($\Theta + \Psi$) (rad)
128	0.0474 + 0.1190	0.0595 + 0.2186
64	0.0527 + 0.1348	0.0662 + 0.2509
32	0.0583 + 0.1542	0.0732 + 0.2875
16	0.0572 + 0.1495	0.0717 + 0.2799
8	0.0576 + 0.1515	0.0724 + 0.2819

## Discussion and conclusion

We implemented a generic vectorial hologram strategy using a single-structure cross-shaped metasurface, enabling it to generate predefined vectorial holograms under specific polarized-light illumination. Its operational wavelength range is extended to the visible light region at 633 nm. This wavelength band is compatible not only with classic helium–neon lasers but also with mainstream commercial optical devices such as semiconductor laser diodes (LDs) and superluminescent light-emitting diodes (SLEDs). This significantly enhances the practicality and integration potential of the technology. This method features high integration, ultra-thin thickness, and is applicable to arbitrary incident polarization.

Subsequently, we employed this approach to design two vectorial holographic metasurfaces and to perform full-wave simulations. We also simulate the intensity results after passing through polarizers with different angles. The simulation results confirmed that the generated holographic images exhibited the expected distribution in both intensity and polarization.

The study may inspire new possibilities in polarization optics, such as polarization-encoded optical storage, dynamic polarization-controlled displays, and polarized illumination, thereby advancing their application in ultra-compact polarization optical systems.

## Data Availability

The raw/processed data required to reproduce these findings can be requested from the corresponding author.
